# STRENGTH IN AGE-FRIENDLY HEALTH SYSTEMS: AN INNOVATIVE INTEGRATED INTERPROFESSIONAL MODEL

**DOI:** 10.1093/geroni/igz038.3054

**Published:** 2019-11-08

**Authors:** Teri Kennedy

**Affiliations:** The University of Kansas Medical Center, Kansas City, Kansas, United States

## Abstract

This paper presents an innovative conceptual approach to health care policy for older adults: the Age-Friendly Health Systems Integrated Interprofessional Model. In 2017, the John A. Hartford Foundation and Institute for Healthcare Improvement, in partnership with the American Hospital Association and Catholic Health Association of the United States, advanced the concept of an Age-Friendly Health System. This initiative is designed to respond to the needs of a burgeoning U.S. older adult population, expected to double from 2012 to 2050, largely due to the aging of Baby Boomers and increased life expectancy. These Baby Boomers will demand a well-coordinated, communicative health system responsive to their values and preferences. In an Age-Friendly Health System, all older adults receive the best possible care, without care-related harms, and with satisfaction of care received. Essential elements include what matters, mentation, mobility, and medications, with a focus on patient-directed, family-engaged care. While a solid framework for improving healthcare for older adults, this model is further strengthened by incorporating the essential elements of person-, family-, and community-centered approaches to care; interprofessional team-based competencies, and Quadruple Aim outcomes. This enhanced model, referred to as the Age-Friendly Health System Integrated Interprofessional Model, combines elements essential to quality healthcare within the framework of an Age-Friendly Health System. This paper will present the original Age-Friendly Health System framework, the proposed Age-Friendly Health System Integrated Interprofessional Model, then compare and contrast each model’s essential principles. Implications for adoption of this enhanced model for policy, education, and practice will be explored.

